# Identification of novel modulators for ionotropic glutamate receptor, iGluA2 by in-silico screening

**DOI:** 10.1186/1742-4682-10-46

**Published:** 2013-07-15

**Authors:** Balasundaram Padmanabhan

**Affiliations:** 1 Department of Biophysics, National Institute of Mental Health and Neuro Sciences (NIMHANS), Bangalore 560 029, India

**Keywords:** Ionotropic glutamate receptors, Neurological disorders, iGluA2, Modulators, Virtual screening, New hit compounds

## Abstract

**Background:**

Ionotropic glutamate receptors (iGluAs, IUPHAR nomenclature) are the major excitatory amino acid neurotransmitter receptors in the mammalian central nervous system (CNS). iGluAs are potential therapeutic drug targets for various neurological disorders including ischemia, epilepsy, Parkinson’s and Alzheimer’s diseases. The known iGluA modulators, cyclothiazide (CTZ), IDRA-21, and other benzothiadiazide derivatives (ALTZ, HCTZ, and CLTZ) bind to the ligand-binding domain of flip-form of iGluA2 at the dimer interface, thereby increasing steady-state activation by reducing desensitization.

**Methods:**

To discover new modulator compounds, we performed virtual screening for the ligand binding domain (LBD) of iGluA2 against NCI Diversity Set III library containing 1597 compounds, and subsequently performed binding-energy analysis for selected compounds. The crystal structure of rat iGluA2 S1S2J (PDB ID: 3IJO) was used for docking studies.

**Results and conclusion:**

From this study, we obtained four compounds: (1) 10-2(methoxyethyl)-3-phenylbenzo[g]pteridine-2,4-dione, (2) 2-benzo[e]benzotriazol-2-yl-aniline, (3) 9-nitro-6H-indolo-(2,3,-b)quinoxaline, and (4) 1-hydroxy-n-(3-nitrophenyl)-2-napthamide. The binding mode of these four compounds is very similar to that of abovementioned established modulators: two molecules of each compound independently bind to the protein symmetrically at the dimer interface; occupy the subsites B, C, B’ and C’; potentially interact with Ser518 and Ser775. Binding energy analysis shows that all the four hits are comparable to the drug molecule, CTZ, and hence, we propose that the discovered hits may be potential molecules to develop new chemical libraries for modulating the flip form of iGluA2 function.

## Background

Ionotropic glutamate receptors (iGluAs) are a family of ligand-gated ion channels that are primarily localized to chemical synapses. They mediate fast excitatory neurotransmission in the mammalian central nervous system (CNS) [1,2] and references therein. Based on sequence homology and pharmacology, iGluAs are classified into four subfamilies, namely, AMPA, Kainate, NMDA and δ-receptors. iGluAs are critical for normal operations of cellular and synaptic activity and plasticity. Dysregulation of these ion channels is frequently linked to the pathogenesis of a wide range of neurological disorder. For example, dysregulation of AMPA receptors leads to various chronic neuronal diseases such as depression, epilepsy, multiple sclerosis, Parkinson’s and Alzheimer’s diseases [3]. Also, Ischemia (stroke) leads to dysregulation of AMPA receptors. Neuronal death, called excitotoxicity, is induced by excessive stimulation of neuronal glutamate receptors. Ischemia is the most common cause of neuronal activation that induces large increases in glutamate release [4]. It has been clearly shown that inhibiting glutamate receptors using AMPA receptor antagonists can attenuate ischemic injury of neuronal tissue in animal experiments [5]. It has also been demonstrated that the non-competitive AMPA antagonists, in cell cultures, could prevent glutamate-induced neuronal death at any agonist concentration, whereas the protective effect of competitive AMPA antagonists was lost at high agonist concentration [6]. Therefore, inhibition of AMPA receptors using non-competitive method can be more beneficial for treatment than inhibition of AMPA receptors in a competitive manner. Positive allosteric modulators improve short-term memory in humans by slowing-down the deactivation of AMPA receptors [7], and hence these kinds of modulators may be beneficial for the treatment of depression and other disorders and diseases of CNS [3,8].

**Figure 1:**
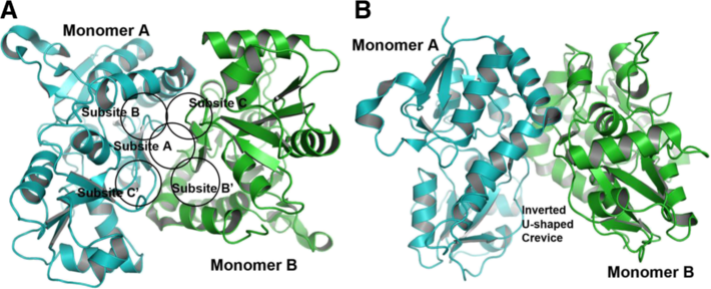
**The cartoon structure of iGluA2-LBD (lig and binding domain) dimer. (A)** Showing the dimer interface region (in arbitrary orientation), and the five subsites A, B, C, B’ and C’ in the ligand binding-site region as defined by Ptak et al. [10]. **(B)** Orthogonal view with respect to Figure 1A to show inverted U-shaped crevice. Two monomers are colored in cyan and green. All cartoon figures were produced with the PyMOL program (http://www.pymol.org), unless otherwise mentioned.

The iGluA protein is a tetramer (dimer of dimers), and each subunit can further be divided into four discrete domains: the extracellular amino-terminal domain (ATD), the extracellular ligand-binding domain (LBD), the transmembrane domain (TMD) and an intracellular carboxy-terminal domain (CTD). The highly conserved LBD structure adopts a clamshell-like conformation. The LBDs of AMPA receptors form a dimer of dimers with a two-fold symmetry, whereas the tetrameric channel-forming domain is assembled with four-fold symmetry [9]. The dimer interface of the LBD of iGluA2 (a subtype of AMPA receptor) forms an inverted U-shaped crevice with two-fold symmetry (Figure 1).

Three structural classes of positive AMPA receptor modulators are available as of now: 1) pyrrolidinone and related piperidine compounds (e.g., aniracetam, CX614, CX516, CX516), 2) benzothiadiazide derivatives (e.g., IDRA-21 and S18986), and 3) biarylpropylsulfonamide compounds (e.s., PEPA, LY404187). The LBD-GluA2– cyclothiazide (CTZ) complex was the first crystal structure which described how an allosteric modulator inhibits desensitization through interacting with LBD of iGluA2 [11]. The CTZ compound is moderately selective for the flip-form of AMPA receptors [12]. Subsequently, structures of several other allosteric modulators complexed with LBD of GluA2 have been determined [13-15]. Moreover, crystal structures of the iGluA2-LBD complexed with benzothiadiazide derivatives (IDRA-21, hydroflumethiazide, hydrochlorothiazide, chlorothiazide, trichlormethiazide, and althiazide) were recently determined for the flip-form of AMPA receptors [10].

To find the structurally diverse potential compounds, we performed virtual screening for LBD of iGluA2 against NCI Diversity Set III containing 1597 compounds. Here, we discuss four compounds which could be potential allosteric modulators for the flip-form of iGluA2: 10-(2-methoxyethyl)-3-phenylbenzo[g]pteridine-2,4-dione, 2-benzo[e]benzotriazol−2-yl-aniline, 1-hydroxy-N-(3-nitrophyenyl)-2-napthamide, and 9-nitro-6H-indolo-(2,3,-b) quinoxaline.

## Methods

### Virtual screening calculations

The AutoDock Vina 1.1.2 [16] was used for virtual screening. The crystal structure of rat iGluA2 S1S2J (lig and binding domain: N392-K506 and P632 – S775; PDB ID: 3IJO) [10] was used as a model for the docking procedure. The docking protocol was set to rigid condition and the size of the dock grid 21 Å × 17 Å × 25 Å, which encompassed the dimer interface of iGluA2-LBD. Exhaustiveness was set to 20 with all other parameters set on default values. About 1597 structurally diversified compounds of the NCI Diversity Set III were used for virtual screening. All calculations were done on the Intel core i5 processor and 4 GB of RAM running on the Ubuntu 12.04 operating system. The top-ranked compounds, sorted by binding energy values, were visually inspected for good chemical geometry and docking. For visualization, docking poses generated by AutoDock Vina were directly loaded into PyMol (http://www.pymol.org) through PyMOL Autodock/Vina Plugin [17]. Pictures of the modeled protein-ligands complex were produced by PyMol.

### Docking energy analysis

To further confirm the docking results, another robust program, DSX (DrugScore eXtented) [18] was used to estimate the binding energy of the ligands bound to the LBD of iGluA2. DSX uses a knowledge-based scoring function based on the DrugScore formalism [19]. The ligand with the larger negative score has a theoretical higher affinity.

## Results and discussion

The initial step in activation of glutamate receptor is the binding of the agonist (glycine, D-serine, aspartate, and glutamate analogues) to the ligand-binding domain (LBD) of the receptor. The LBD comprises a dimer of dimers relative to the more symmetrical assembly of the tetramer channel-forming domain. The dimer interface of the iGluA2-LBD forms an inverted U-shaped crevice with two-fold symmetry (Figure 1). At the dimer interface, as defined by Ptak et al. [10], the binding site region can be subdivided into five overlapping subsites (Figure 1A). The central subsite (subsite A) and two symmetrical copies of two subsites (subsites B, C, B’ and C’) are bordered by residues from each of the two LBD chains.

**Figure 2:**
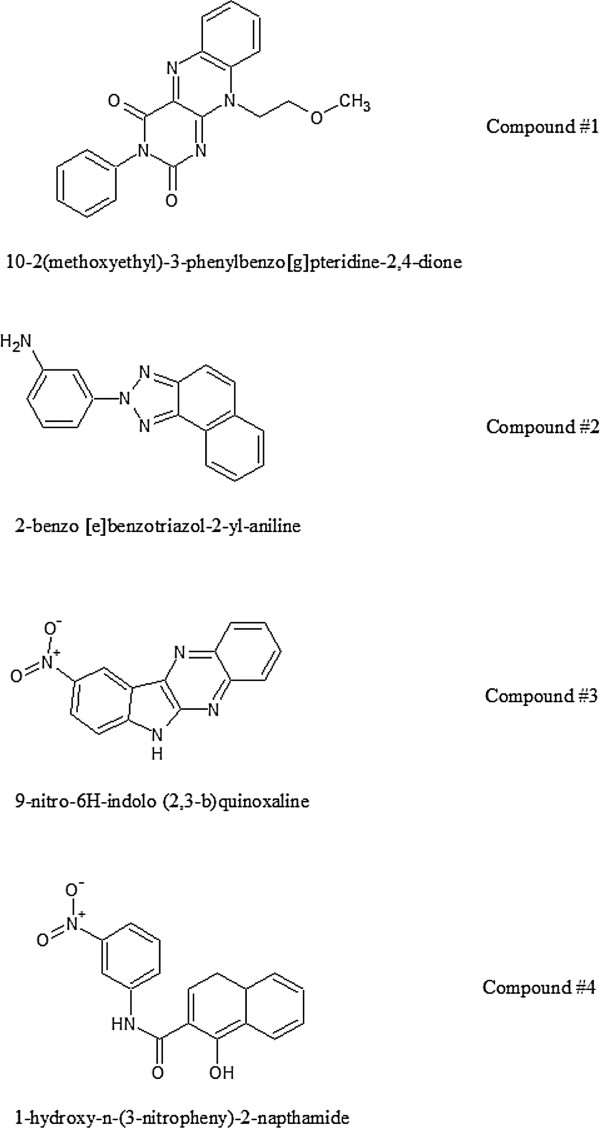
**Chemical structures of hit compounds.** Structures were produced with ACD/ChemSketch (http://www.acdlabs.com).

The virtual screening results, obtained from AutoDock Vina, were sorted on the basis of their predicted binding free energies (ΔG_vina_). The predicted binding conformations for the selected compounds from this pool were visually checked using PyMol plugged with Autodock Vina [17]. From this analysis, we observed four potential compounds bind well to the iGluA2-LBD at the dimer interface (Figure 2).

### Compound #1: 10-(2-methoxyethyl)-3-phenyl benzo [g] pteridine-2,4-dione

**Figure 3:**
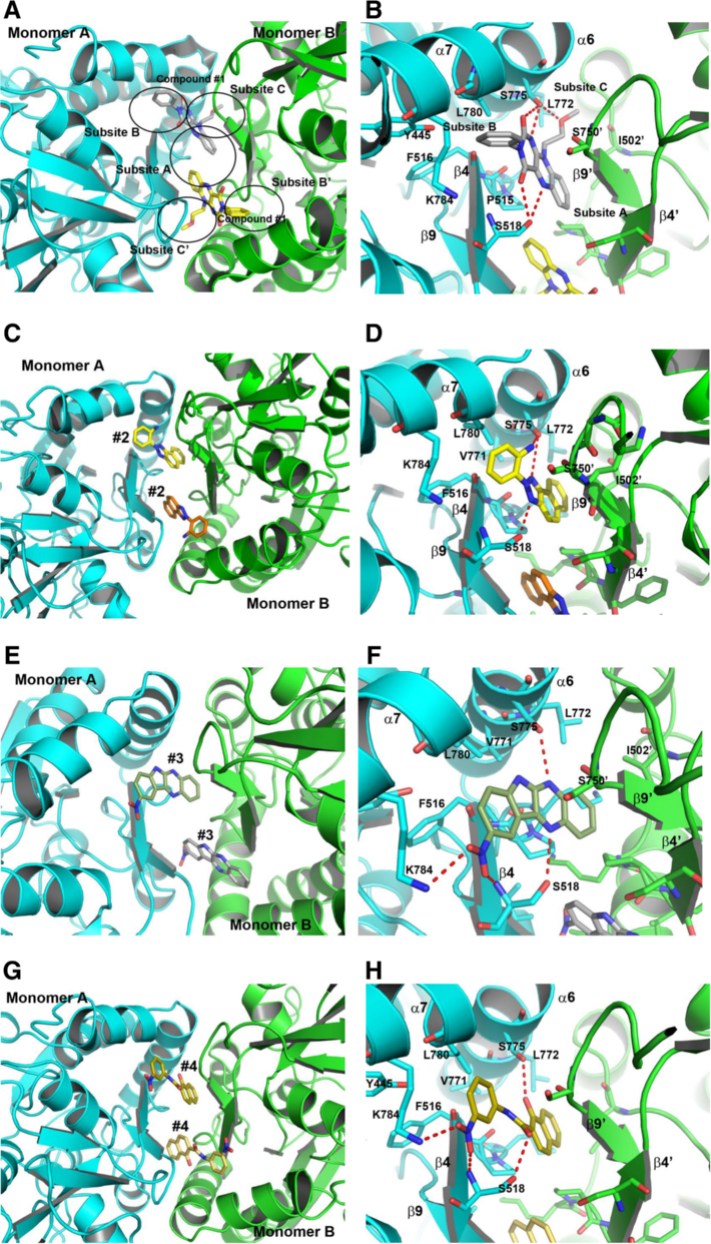
**Binding of compounds #1–4 with the iGluA2-LBD dimer.** Two molecules of each compound independently bind at the dimer interface are shown in **(A)**, **(C)**, **(E)** and **(G)** for the compounds #1, #2, #3, and #4, respectively. The close-view of the corresponding compounds to show protein-ligand interactions are shown in **(B)**, **(D)**, **(F)** and **(H)**, respectively. For compound #1, the five subsites which are contributing protein-ligand interactions are shown in circles. The ligands and interacting residues are shown in sticks. The oxygen and nitrogen atoms are colored in red and blue, respectively.

The compound #1, 10-(2-methoxyethyl)-3-phenyl benzo [g] pteridine - 2,4-dione, binds to the LBD of iGluA2 at the dimer interface. The two molecules of #1 bind to iGluA2 independently: one at the B & C subsites, and the other one binds to B’ & C’ symmetry subsites (Figures 3A,B). The central subsite A also shares interaction with the compounds; however, it possesses less interaction compared to that found in the subsites B & C. The cyclic 3-rings structure of the compound is sandwiched between the β-strand (β9’: Lys751 – Tyr753) of chain B and part of a long-loop connecting the β-strand, β4 of the other chain A (Lys514 – Phe516) (Figure 3B). The sandwiched moiety is stabilized by hydrophobic interactions. The phenyl ring is hydrophobically interacting with Leu780 in the subsite B. The benzene group binds partially in the central subsite A. An oxygen atom of 2,4-dione is hydrogen bonded to Ser775 which lies in the α-helix, α6, while the other oxygen atom of 2,4-dione contributes a hydrogen bond to Ser518. A nitrogen atom of pteridine moiety shares a hydrogen bond with Ser775. The methoxy ethyl moiety is positioned in the subsite C peripheral region whereas the phenyl group is well placed in the peripheral region of the subsite B. The subsite C is predominantly hydrophobic which is contributed by Leu772, Lys514 (aliphatic carbon atoms of the side chain), and I502’. Besides hydrophobic interactions in this region, the oxygen atom of methoxy group makes a hydrogen bond to Ser775. The second molecule also possesses similar kind of interactions with the subsites B’ and C’.

### Compound #2: 2-benzo [e] benzotriazol-2-yl-aniline

Two molecules of #2 independently bind to iGluA2 as observed in compound #1 (Figures 3C,D). The three-membered ring is sandwiched between the loop connecting the β-strand, β4 in chain A and β-strand, β9’ in chain B. The polar benzotriazole group of three-membered cyclic ring makes hydrogen bonds with Ser518 and Ser775 (Figure 3D). Moreover, Ser775 contributes another hydrogen bond to a nitrogen atom of aniline moiety which sits in the subsite B. The aniline group also possesses hydrophobic interactions with Leu780. In this complex, the subsite C is partially occupied by a non-polar naphthalene group. Incorporating a small hydrophobic group by connecting naphthalene moiety may contribute additional binding affinity with the protein. We observed that the subsite A is free from ligand contacts.

### Compound #3: 9-nitro-6H-indolo (2,3,-b) quinoxaline

In this complex, the rigid cyclic ring structure of indolo-quinoxaline group is sandwiched at the dimer interface, and occupying subsites B and C (Figures 3E,F). The quinoxaline group is positioned in the subsite C. The hydroxyl group of Ser775 is hydrogen bonded to a nitrogen atom of quinoxaline. The Ser518 residue contributes hydrogen bond interactions with the nitro group and another nitrogen atom of quinoxaline through its main-chain and side-chain, respectively (Figure 3F). The nitro group also makes a potential salt bridge with Lys784 which is positioned in the subsite B. The subsite A is less occupied from ligand binding. The second molecule also binds in a similar mode to the symmetry subsites, B’ and C’ of the LBD domain.

### Compound #4: 1-hydroxy-N-(3-nitrophenyl)-2-napthamide

In this complex, two molecules are independently sandwiched between the two monomers of the β-strand, β9’ and a loop connecting the β-strand, β4 (Figures 3G,H). The naphthalene moiety binds to the subsite C. The hydroxyl group of naphthalene moiety shares a hydrogen bond with Ser775. The subsite B is occupied by the nitrophenyl group. The phenyl ring makes hydrophobic interactions with Leu780. Ser518 contributes two hydrogen bonds with carbonyl and nitro-groups (Figure 3H). The nitro-group contributes additional salt bridge with Lys784. The subsite A is free from ligand interactions.

### Comparison between compounds #1–4

**Figure 4:**
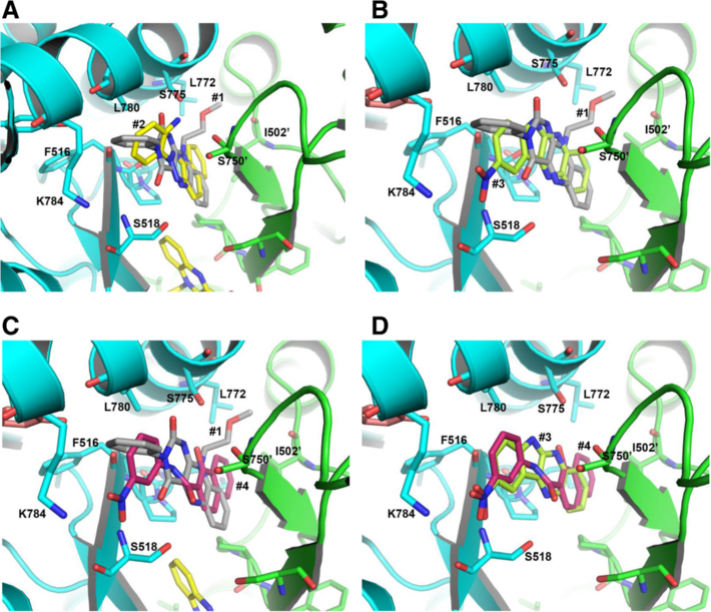
**Binding comparisons between the compounds #1–4.** Superposition of compound **(A)** #1 onto #2, **(B)** #1 onto #3, **(C)** #1 onto #4, and **(D)** #3 onto #4. The ligands and interacting residues are shown in sticks. The oxygen and nitrogen atoms are colored in red and blue, respectively.

We observed intriguing results when compared the compounds #1–4 by superpositioning theirs docking structures onto each other (Figure 4). The hydrophobic subsite C is substantially occupied by the compound #1 compared to other compounds suggesting that aliphatic group in that region for compounds #2–4 may enhance the affinity for the protein interaction. For the predominant hydrophilic subsite B region, compounds #3 and #4 possess considerable affinity through both hydrophilic and hydrophobic interactions (Figures 4C,D) compared to the compounds #1 and #2 (Figure 4A). For compounds #1 and #2, incorporating polar groups by linking with phenol and aniline, respectively, to make hydrophilic interactions with Lys784 and Ser518 may significantly increase their affinity for the iGluA2-LBD binding. Another interesting feature is also observed that Ser518 and Ser775 are consistently make hydrogen bond interactions with all the four compounds suggesting their critical role for ligand binding. Among these four compounds, all the three compounds except compound #1 possess less interactions in the A subsite.

### Comparison of compounds #1–4 with the other AMPA modulators

The known positive AMPA receptor modulators fall into three structural classes, namely: 1) pyrrolidinone and related piperidine compounds (e.g., aniracetam, CX614, CX516, CX516), 2) benzothiadiazide derivatives (e.g., IDRA-21 and S18986), and 3) biarylpropylsulfonamide compounds (e.g., PEPA, LY404187). The first crystal structure of the iGluA2-LBD complex was solved using the compound, cyclothiazide (CTZ) [11]. The CTZ compound is moderately selective for the flip-form of AMPA receptors [12]. Subsequently, structures of several other allosteric modulators complexed with LBD of iGluA2 have been determined [13-15]. Moreover, crystal structures of the iGluA2-LBD complexed with benzothiadiazide derivatives (IDRA-21, hydroflumethiazide, hydrochlorothiazide, chlorothiazide, trichlormethiazide, and althiazide) were recently determined for the flip-form of AMPA receptors [10].

**Figure 5:**
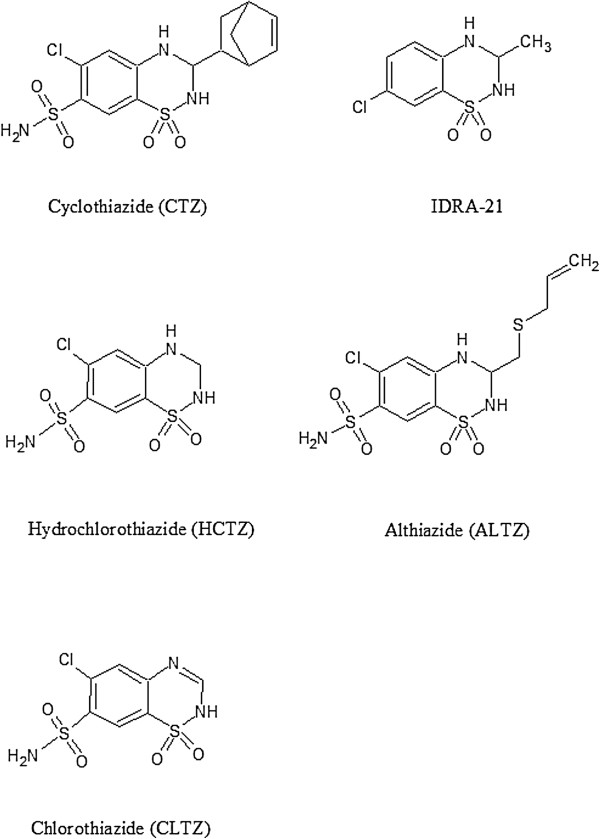
Chemical structures of CTZ, IDRA-21, HCTZ, ALTZ and CLTZ modulators.

**Figure 6:**
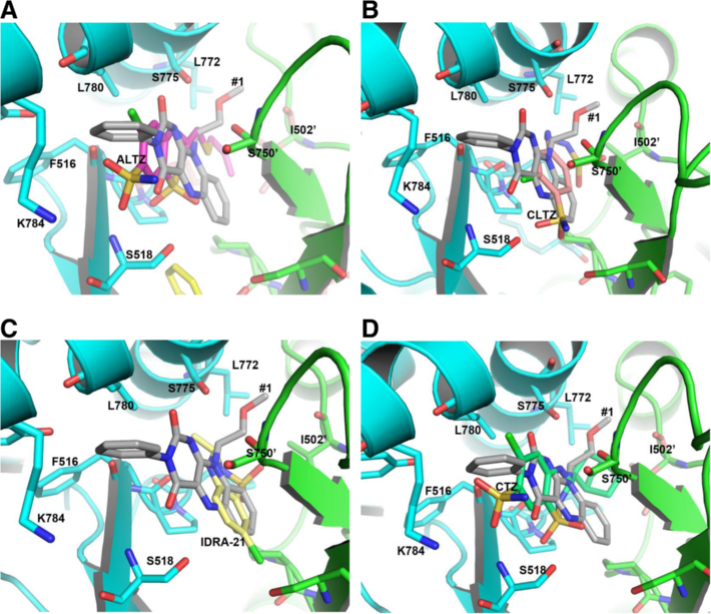
**Binding comparison between the compound #1 and the known modulators whose crystal structures were already known.** Superposition of compound #1 onto **(A)** ALTZ (PDB ID: 3IJO), **(B)** CLTZ (PDB ID: 3IK6), **(C)** IDRA-21 (PDB ID: 3IL1), and **(D)** CTZ (PDB ID: 1LBC). The ligands and interacting residues are shown in sticks. The oxygen and nitrogen atoms are colored in red and blue, respectively.

We analyzed our predicted structures by comparing with few compounds whose crystal structures were already determined (Figures 5 and 6). The benzothiadiazide derivative structures were recently reported by Ptak et al. [10]. The ALTZ and TCMZ molecules bind to the subsites B and C. As shown in Figure 6A, the subsite C is significantly occupied by both the compounds #1 and ALTZ (PDB ID: 3IJ0) through their aliphatic group. When compared the compound #1 with the CLTZ structure (PDB ID: 3IK6), CLTZ occupy the subsite C and partially to the central subsite A, suggest the importance of C-subsite hydrophobic pocket (Figure 6B). The other derivative HCTZ (PDB ID: 3IJX) also possesses similar structural features (not shown). The IDRA-21 structure (PDB ID: 3IL1) showed that the IDRA-21 ligand occupies the central subsite A and partially to the subsite C by methyl group (Figure 6C). The CTZ (cyclothiazide) structure (PDB ID: 1LBC) revealed that the CTZ molecule binds to the B & C subsites makes the modulator extremely potent; but, lacking ability to cross the blood–brain barrier emphasis a need for designing drugs that can target AMPA receptors *in vivo*[11,15]. In all these structural analysis, it highlights the important residues Ser518 and Ser775 for potential hydrophilic interactions in the flip form of iGluA2 subtype. All the compounds #1–4 unambiguously interact with Ser518 and Ser775 as observed in the known crystal structures of modulators suggest the predicted compounds may indeed potential ligands for iGluA2 binding.

### DSX analysis

To further confirm our predicted compounds are potential ligands for iGluA2 binding, we used another robust program, DSX (DrugScore eXtented) [18] to perform binding energy analysis of the ligands bound to the LBD of iGluA2. DSX uses a knowledge-based scoring function based on the DrugScore formalism [19]. The ligand with the larger negative score has the higher affinity. We carried out this analysis for the compounds #1–4 as well as for the known well-studied compounds, ALTZ, HCTZ, CLTZ, IDRA-21 and CTZ which occupy the B & C subsites in the LBD complex (Table 1). The binding score value for cyclothiazide (CTZ) is −127, and that for ALTZ, HCTZ, CLTZ and IDRA-21 is −65, -65, -48, and −74, respectively. Intriguingly, the binding score values for predicted compounds #1–4 are greatly comparable to that for CTZ. The compounds #1, #2 and #4 possess −126, -121 and −120, respectively, and the compound #3 possesses −107. Hence, we speculate from our present analysis that all our predicted compounds #1–4 may indeed potential modulators for the flip-form of iGluA2, and may also be potential initial hits to develop new chemical libraries to regulate iGluA2 function.

**Table 1:** Docking energy analysis by DSX procedure

**Ligand**	**Score**
ALTZ	−65.0
IDRA-21	−74.53
HCTZ	−64.62
CLTZ	−48.36
CTZ	−127.40
Compound 1	−126.20
Compound 2	−121.43
Compound 3	−107.35
Compound 4	−120.45

## Conclusion

Ionotropic glutamate receptors are the major excitatory amino acid neurotransmitter receptors in the vertebrate central nervous system (CNS). AMPA receptors, a subfamily of iGluAs, mediate the majority of the fast excitatory amino acid synaptic transmission in the CNS. Discovering modulators to regulate the function of AMPA receptors may provide numerous therapeutic avenues in the field of CNS drug discovery. In this aspect, we have discovered four compounds from virtual screening and docking studies using NCI Diversity Set III library containing 1597 compounds. All of these compounds, 1–4 bind to the dimer interface of iGluA2-LBD, and the binding mode of these compounds are essentially similar to the known established compounds such as cyclothiazide (CTZ), IDRA-21 and benzothiadiazide derivatives of ALTZ, HCTZ, CLTZ and HCTZ. Moreover, the binding energy analysis using DSX method revealed that all the predicted four compounds are unexpectedly comparable to the potential drug molecule, CTZ and much better than IDRA-21 and benzothiadiazide derivatives. Hence, we speculate that predicted molecules (compounds 1–4) are potential hits to develop new chemical libraries as modulators for AMPA receptors.

## Competing interests

The author declares that he has no competing interests.
